# Bioactive Compounds, Technological Advances, and Sustainable Applications of Avocado (*Persea americana* Mill.): A Critical Review

**DOI:** 10.3390/foods14152746

**Published:** 2025-08-06

**Authors:** Amanda Priscila Silva Nascimento, Maria Elita Martins Duarte, Ana Paula Trindade Rocha, Ana Novo Barros

**Affiliations:** 1 Academic Unit of Food Engineering, Federal University of Campina Grande, Av. Aprígio Veloso, 882, Campina Grande 58429-900, PB, Brazil; melitamd@gmail.com (M.E.M.D.); ana_tridade@yahoo.com.br (A.P.T.R.); 2 Centre for the Research and Technology of Agro-Environmental and Biological Sciences (CITAB), University of Trás-os-Montes e Alto Douro (UTAD), 5000-801 Vila Real, Portugal

**Keywords:** *Persea americana*, bioactive lipids, green extraction technologies, functional foods, phytochemicals, phytosterols, nanostructured systems, precision nutrition, nutraceuticals, cosmeceuticals

## Abstract

Avocado (*Persea americana*), originally from Mesoamerica, has emerged as a focus of intense scientific and industrial interest due to its unique combination of nutritional richness, bioactive potential, and technological versatility. Its pulp, widely consumed across the globe, is notably abundant in monounsaturated fatty acids, especially oleic acid, which can comprise over two-thirds of its lipid content. In addition, it provides significant levels of dietary fiber, fat-soluble vitamins such as A, D, E and K, carotenoids, tocopherols, and phytosterols like β-sitosterol. These constituents are consistently associated with antioxidant, anti-inflammatory, glycemic regulatory, and cardioprotective effects, supported by a growing body of experimental and clinical evidence. This review offers a comprehensive and critical synthesis of the chemical composition and functional properties of avocado, with particular emphasis on its lipid profile, phenolic compounds, and phytosterols. It also explores recent advances in environmentally sustainable extraction techniques, including ultrasound-assisted and microwave-assisted processes, as well as the application of natural deep eutectic solvents. These technologies have demonstrated improved efficiency in recovering bioactives while aligning with the principles of green chemistry. The use of avocado-derived ingredients in nanostructured delivery systems and their incorporation into functional foods, cosmetics, and health-promoting formulations is discussed in detail. Additionally, the potential of native cultivars and the application of precision nutrition strategies are identified as promising avenues for future innovation. Taken together, the findings underscore the avocado’s relevance as a high-value matrix for sustainable development. Future research should focus on optimizing extraction protocols, clarifying pharmacokinetic behavior, and ensuring long-term safety in diverse applications.

## 1. Introduction

Avocado (*Persea americana* Mill.), a fruit native to Mesoamerica, has garnered increasing interest due to its notable nutritional, functional, and technological attributes. Now widely cultivated across tropical and subtropical regions, its pulp is particularly rich in monounsaturated fatty acids—especially oleic acid, which comprises approximately 67% to 71% of its total lipid content—as well as soluble fibers, fat-soluble vitamins (A, D, E, and K), tocopherols, carotenoids, and phytosterols such as β-sitosterol [1]. This distinctive composition underpins the fruit’s antioxidant, anti-inflammatory, hypoglycemic, and cardioprotective properties, as demonstrated in both clinical and experimental studies.

From an epidemiological perspective, data from a nationally representative cross-sectional Australian survey revealed that avocado consumers had significantly lower body mass index, waist circumference, and systolic blood pressure, alongside improved intakes of fiber, monounsaturated fats, and essential micronutrients compared to non-consumers [2]. Supporting this, controlled clinical trials—such as the one by Zhang et al. [3]—have shown that the daily consumption of one whole avocado over 12 weeks significantly reduced total cholesterol and improved cardiometabolic profiles in individuals with insulin resistance.

In parallel, emerging technologies have been increasingly applied to enhance the extraction of avocado’s bioactive constituents. Green extraction techniques—including ultrasound-assisted extraction (UAE), microwave-assisted extraction (MAE), and the use of natural deep eutectic solvents (NADES)—have shown superior efficiency over conventional methods, both in extraction yield and in preserving the functional integrity of compounds. These sustainable approaches are consistent with the principles of green chemistry and the circular bioeconomy, representing innovative pathways for the development of functional foods, cosmeceuticals, and pharmaceutical applications [4,5].

Among commercial cultivars, Hass is the most widely cultivated due to its high oil content, extended shelf life, and superior sensory attributes, while others such as Fuerte and Bacon are valued regionally for their adaptability and milder flavor profiles [1,2]. Parallel to its economic expansion, there is a rising scientific interest in understanding the compositional diversity and health effects of different avocado parts—including pulp, peel, and seed—stimulating innovation in food, nutraceutical, and cosmetic applications [1,3,6,7]. Moreover, the incorporation of avocado and its derivatives into processed foods has been explored in recent formulations such as low-fat muffins [4,5] and functional dressings [6], reinforcing its role as a versatile and sustainable raw material in health-oriented product development.

In this context, this review aims to critically synthesize current scientific knowledge on the nutritional composition, bioactive potential, technological advances, and sustainable applications of avocado (*Persea americana*) [1]. Special emphasis is given to green extraction technologies, innovative strategies for bioactive compound delivery, and their integration into functional food development and personalized nutrition systems. By outlining these aspects, this work seeks to identify current challenges, highlight knowledge gaps, and propose future research directions to enhance the value and application of avocado in health-promoting and sustainable products.

**Figure 1:**
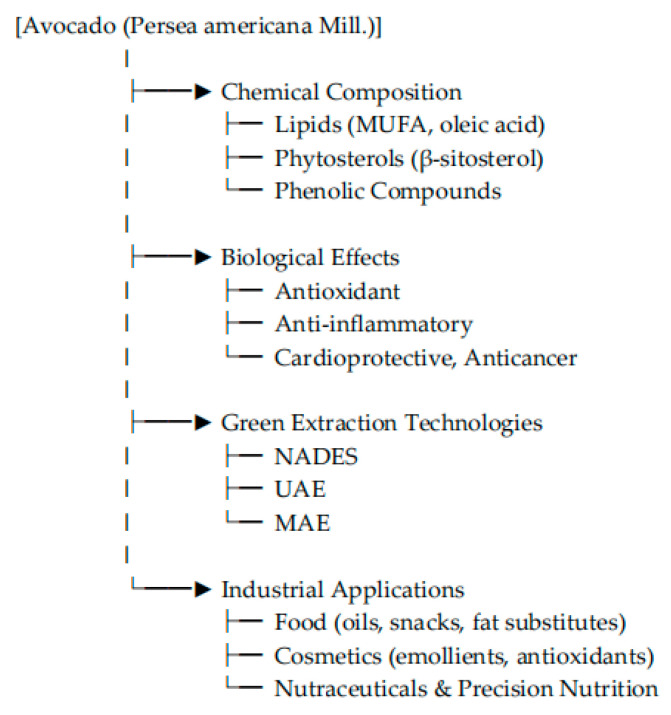
Conceptual diagram of the thematic scope of this review on *Persea americana*.

To guide the reader through the structure of this review, a conceptual diagram summarizing its thematic scope is provided in Figure 1.

For the development of this critical review, publications indexed in major scientific databases (PubMed, ScienceDirect, Scopus, and Web of Science) were selected, with a primary focus on original studies published between 2020 and 2025. The search strategy employed descriptors such as “avocado”, “*Persea americana*”, “bioactive compounds”, “green extraction”, “antioxidant activity”, and “technological applications”. Review articles were included in a complementary manner, only when essential to support broader concepts, consolidate theoretical frameworks, or present updated overviews. The final selection prioritized studies with methodological rigor, thematic relevance, and alignment with the scope of this work.

## 2. Chemical and Phytochemical Composition: Current State of Scientific Knowledge

Avocado (*Persea americana* Mill.) is widely acknowledged for its unique nutritional and phytochemical profile, particularly its abundance of unsaturated lipids, phenolic compounds, and other bioactive secondary metabolites. These components have demonstrated therapeutic potential at cellular, metabolic, and clinical levels, reinforcing the classification of avocado as a valuable functional food. Nonetheless, despite the increasing volume of scientific research, the phytochemical characterization of this fruit—especially among less common or region-specific cultivars—remains incomplete and warrants further investigation.

### 2.1. Lipid Profile and Predominant Functional Compounds

Avocado pulp is among the richest plant-based sources of lipids, exhibiting a lipid profile considered highly beneficial to human health. Depending on the cultivar and ripeness stage, lipids may represent approximately 15% to 30% of the fresh pulp mass. Detailed analyses of the Hass variety indicate that total lipids account for about 19.7% of fresh weight, with oleic acid (C18:1) as the predominant fatty acid, followed by palmitoleic (C16:1) and linoleic (C18:2) acids [6]. Most of these lipids are monounsaturated fatty acids (MUFA), with oleic acid (C18:1 n-9) contributing between 60% and 71% of total fatty acids [1].

Beyond oleic acid, minor components such as palmitic (C16:0), linoleic (C18:2 n-6), stearic (C18:0), and α-linolenic (C18:3 n-3) acids contribute to a lipid profile that supports cardiovascular health. The physiological benefits associated with avocado consumption are largely attributed to these fatty acids—especially oleic acid—which has been extensively investigated for its anti-inflammatory and hypocholesterolemic effects, as well as its capacity to modulate gene expression related to lipid metabolism [3].

Evidence from randomized clinical trials and systematic reviews further reinforces the role of daily avocado intake in improving lipid and glycemic profiles. In a comprehensive systematic review, Permal et al. [7] analyzed 45 clinical and observational studies, reporting consistent reductions in low-density lipoprotein (LDL), particularly small, dense and oxidized LDL particles, and triglycerides. Concurrently, several studies noted increases in high-density lipoprotein (HDL), especially in the concentration of larger and more functional HDL subfractions—particularly in individuals with overweight or cardiometabolic risk.

Phytosterols are another relevant class of bioactive lipids found in avocado pulp, with β-sitosterol being the most abundant. These compounds, particularly β-sitosterol, have been associated with cholesterol-lowering and anti-inflammatory effects, contributing to the fruit’s overall cardioprotective properties [1].

The antioxidant potential of avocado oil is further enhanced by its tocopherol content, particularly α-tocopherol (vitamin E), detected at concentrations up to 24.5 mg/100 g [8]. This compound plays a key role in protecting cellular membranes from lipid peroxidation.

From a technological standpoint, avocado oil exhibits high thermal stability. In a comparative analysis, Berasategi et al. [8] reported higher phytosterol content in avocado oil (339.6 mg/100 g) compared to olive oil (228.3 mg/100 g). Moreover, avocado oil retained its physicochemical properties after heating at 180 °C for up to nine hours, demonstrating oxidative resistance comparable to that of olive oil.

Despite the substantial data available for commercial cultivars such as Hass and Fuerte, detailed investigations into regional Latin American varieties remain scarce. The lipid composition of these underexplored cultivars may vary significantly due to differences in genotype, climate, soil, and cultivation practices. This knowledge gap constrains the full nutritional and economic valorization of native varieties in countries such as Brazil, Colombia, and Mexico.

### 2.2. Phenolic Compounds and Antioxidant Activity

Phenolic compounds present in avocado have been widely recognized for their role in attenuating oxidative stress and modulating inflammatory pathways. These bioactive metabolites act at both molecular and systemic levels and are considered key contributors to the fruit’s functional health properties.

Avocado pulp contains a broad spectrum of phenolic compounds, with gallic acid, catechin, quercetin, and ferulic acid among the most representative. Recent studies employing advanced analytical techniques have identified up to 64 distinct phenolics in the pulp, including chlorogenic acid and epicatechin, which contribute significantly to the fruit’s antioxidant potential [9,10]. The phenolic profile and concentrations vary depending on the cultivar and ripening stage, with compounds such as gallic acid and quercetin typically present at higher levels in ripe fruits [11]. Catechin and epicatechin, two flavonoids consistently detected in appreciable amounts, are closely linked to the antioxidant activity of the pulp. Ferulic acid and other hydroxycinnamic acids are also present, though their levels tend to fluctuate with fruit maturity [12].

These compounds are well known for their antioxidant, anti-inflammatory, and potential cardioprotective effects. Their presence enhances the nutritional and functional value of avocado, supporting its applicability in food, nutraceutical, and pharmaceutical formulations [2]. Di Stefano et al. [10], in their compositional analysis of six *Persea americana* cultivars, reported substantial levels of phenolic compounds, including gallic acid, catechin, and epicatechin—highlighting their contribution to the overall antioxidant capacity of avocado. Although these concentrations are lower than those found in berries or grapes, they still support the fruit’s functional profile. Moreover, recent studies indicate that phenolic composition is highly influenced by factors such as cultivar, geographic origin, ripening stage, and post-harvest handling [11,12].

Beyond the pulp, phenolic compounds are also abundant in non-edible parts of the fruit, including the peel and seed. These fractions have garnered increasing interest for their potential use in the development of food additives, nutraceuticals, and cosmeceuticals, owing to their high antioxidant activity. The peel, in particular, has been shown to possess significantly higher total phenolic content (TPC) and stronger DPPH and ABTS radical-scavenging capacity than the pulp, highlighting promising opportunities for by-product valorization [13,14,15].

The antioxidant effects of avocado phenolics are not limited to the direct scavenging of reactive oxygen species (ROS); they also involve the modulation of endogenous antioxidant defense systems. Several studies have demonstrated that avocado extracts can upregulate key enzymes such as superoxide dismutase (SOD), catalase (CAT), and glutathione peroxidase (GPx) [16,17]. Moreover, the anti-inflammatory properties of these compounds have been associated with the downregulation of pro-inflammatory mediators, including NF-κB, COX-2, and TNF-α, although most of the evidence remains at the preclinical stage [18,19].

To assess antioxidant capacity, various in vitro methods have been applied, including the DPPH (2,2-diphenyl-1-picrylhydrazyl) radical scavenging assay and the ABTS (2,2′-azino-bis(3-ethylbenzothiazoline-6-sulfonic acid)) cation decolorization assay, as well as ORAC (oxygen radical absorbance capacity), which measures antioxidant inhibition of peroxyl radical-induced oxidation, and FRAP (ferric reducing antioxidant power), which evaluates the ability of antioxidants to reduce ferric ions to ferrous ions. These methods provide complementary insights into different antioxidant mechanisms. These studies consistently demonstrate a moderate but biologically relevant antioxidant potential, particularly in lipid-rich matrices where oxidative stability plays a critical role [20,21].

Despite the growing body of research, further studies are required to comprehensively characterize the phenolic profiles of diverse avocado varieties—particularly underutilized landraces—and to better understand how agricultural practices, processing technologies, and storage conditions influence the retention, stability, and bioavailability of these compounds. Such knowledge is essential for guiding the development of effective functional food products and health-promoting formulations based on avocado [22,23].

### 2.3. Untapped Nutritional and Functional Potential in Lesser-Known Avocado Cultivars

*Persea americana* (avocado) exhibits remarkable diversity across its cultivars, yet much of this variation remains underexplored, especially regarding nutritional and functional properties. Recent studies have highlighted significant differences in the phytochemical profiles, antioxidant capacities, and mineral contents among various avocado cultivars and plant parts, including leaves and seeds [11,12,13,14,15,16,17,18,19,20,21,22,23]. While the Hass variety continues to dominate scientific research and global markets, numerous regional cultivars exhibit distinct—and often superior—profiles in terms of lipids, minerals, and phytochemicals, underscoring their potential value for application in functional foods, cosmetics, and nutraceuticals [24,25,26].

In Morocco, Nasri et al. [27] analyzed eight cultivars, including Maluma Hass and Ettinger, and reported pulp lipid contents of 30.4 g/100 g and 29.0 g/100 g, respectively—values significantly higher than the average 15.0 g/100 g commonly reported for Hass. These cultivars also showed elevated levels of β-sitosterol (up to 4559 mg/kg), as well as calcium (532 mg/kg) and iron (20.3 mg/kg), demonstrating a superior nutritional profile.

In Venezuela, Gómez-López et al. [28] characterized several high-oil avocado cultivars and highlighted the Ryan variety, which exhibited 18.8 g/100 g of pulp lipids alongside favorable sensory and technological characteristics, reinforcing its suitability for functional formulations targeting a health-oriented lipid profile.

Complementing these findings, Oliveira et al. [29] investigated three Brazilian cultivars, Margarida, Breda, and Geada, and reported distinct phytochemical profiles in the peel, seed, and pulp, which were closely associated with antioxidant capacity. Notably, the Breda cultivar exhibited the highest total phenolic content in the seed (83.38 mg GAE/g), highlighting its potential for functional ingredient development. Similarly, Ford et al. [30] conducted a compositional analysis of the Hass variety, confirming its consistent nutritional profile and underscoring its status as a global reference. However, comparative studies such as that of Liu et al. [31], which evaluated genetic diversity and quality traits in Chinese landraces using molecular markers, reveal a broad spectrum of nutritional variation. These findings underscore the importance of preserving and bioprospecting local germplasms to uncover cultivars with enhanced functional and agronomic traits.

More recently, Yang et al. [32] evaluated 95 germplasm accessions and observed considerable variability in nutritional composition. Pulp lipid content ranged from 2.2% to 16.6%, and several genotypes presented elevated levels of essential minerals such as magnesium, iron, and zinc. Based on metabolomic and proximate composition analyses, 14 accessions were identified as superior to the Hass cultivar in at least one nutritional criterion.

Beyond genetic factors, nutritional composition in avocado is strongly modulated by edaphoclimatic conditions—often referred to as “terroir.” Kouam et al. [33], in a study involving 206 avocado accessions from the Bamboos Plateau in western Cameroon, documented wide phenotypic variability influenced by altitude, soil composition, and geographic location. Multivariate and phylogenetic analyses yielded a high diversity index (H′ = 0.90–1.31), highlighting substantial potential for genetic improvement aimed at enhancing functional nutrient density.

Despite the demonstrated compositional and phytochemical richness of these lesser-known cultivars, there is a notable lack of clinical research assessing their physiological and metabolic impacts in humans. Current data are largely limited to in vitro studies and compositional profiling. Future progress in this field will require the design of human clinical trials, bioavailability studies, and extract standardization protocols to support the safe and effective use of these varieties in health-promoting food products.

As summarized in Table 1, cultivars such as Ettinger and Maluma Hass exhibit higher levels of pulp lipids and phytosterols compared to Hass. Although seed composition is beyond the primary scope of this review, some cultivars, such as Breda, have shown exceptionally high concentrations of total phenolic compounds, further emphasizing the underutilized genetic diversity within the *Persea americana* species.

## 3. Functional Studies and Clinical Evidence

Avocado (*Persea americana*) distinguishes itself among tropical fruits due to its complex phytochemical composition and functional lipid profile. A growing body of evidence supports the biological activity of its bioactive constituents—including phenolic compounds, carotenoids, tocopherols, phytosterols, and mono- and polyunsaturated fatty acids—which have demonstrated antioxidant, anti-inflammatory, cardioprotective, and anticancer properties through well-characterized molecular mechanisms [19,31,32,33,34].

These effects result from interactions with molecular targets that regulate oxidative stress and inflammation, such as the transcription factors Nrf2 (nuclear factor erythroid 2-related factor 2), NF-κB (nuclear factor kappa B), and signaling enzymes like LRRK2 (leucine-rich repeat kinase 2), involved in neurodegenerative processes [35,36].

### 3.1. Antioxidant, Anti-Inflammatory, Cardioprotective, and Anticancer Properties

In the clinical context, growing evidence supports the protective role of avocado in cardiovascular health. Li et al. [31] demonstrated, in a randomized clinical trial, that consuming a serving of avocado (68 g) alongside a high-fat meal reduced postprandial inflammation, as measured by lower levels of IL-6 and C-reactive protein (CRP), and preserved endothelial function, assessed by digital pulse amplitude tonometry. This was evidenced by reductions in circulating levels of interleukin-6 (IL-6) and C-reactive protein (CRP), as well as the preservation of endothelial function, assessed via pulse tonometry. These findings are further corroborated by Davis et al. [36], who, in an ancillary study of the Habitual Diet and Avocado Trial (HAT), evaluated endothelial function in adults with abdominal obesity and reported consistent improvements following avocado consumption. Over a 12-week intervention, participants consuming one avocado per day exhibited significant improvements in endothelial-dependent vasodilation, measured by digital pulse amplitude tonometry, compared to the control group following their habitual diet. Moreover, this group showed reductions in vascular stiffness and preserved nitric oxide bioavailability, indicating that daily avocado intake may confer vascular benefits independent of weight loss. These clinical data reinforce the cardioprotective effects of avocado, particularly in populations at elevated metabolic risk. The study also showed that avocado attenuated NF-κB pathway activation by maintaining IκBα protein levels, indicating a direct modulatory effect on acute diet-induced inflammation. These findings are consistent with previous clinical research showing that regular avocado consumption can improve vascular function and modulate cardiometabolic risk factors in overweight individuals [36].

Furthermore, the Habitual Diet and Avocado Trial (HAT), a 26-week longitudinal study, assessed the impact of daily avocado consumption in individuals with abdominal obesity [36]. Matthan et al. [37] reported a modest but significant reduction in serum total and LDL cholesterol levels in the avocado group, without changes in body weight. This suggests that the cardioprotective effects are more related to dietary quality than to weight loss per se.

Additionally, Khan et al. [38] demonstrated that daily avocado consumption over 12 weeks led to a redistribution of visceral adipose tissue and improved postprandial insulin sensitivity in individuals with overweight and obesity. These findings support the potential role of avocado in modulating glycemic responses and abdominal fat deposition, further contributing to its cardiometabolic benefits.

In the same study, Yang et al. [32] found that daily avocado intake positively modulated gut microbiota composition, increasing both alpha and beta diversity, and enriching beneficial genera such as *Faecalibacterium prausnitzii*. This intestinal modulation is relevant due to its association with reduced systemic endotoxemia and chronic low-grade inflammation, acting as a complementary mechanism for cardiometabolic protection.

In the field of experimental oncology, compounds extracted from avocado peel and seed have shown promising antitumor properties. In vitro studies reveal that these extracts induce selective apoptosis in tumor cell lines such as MCF-7 (breast), HT-29 (colon), and HL-60 (leukemia) via caspase activation, DNA fragmentation, and cell cycle arrest.

Additionally, the review by Bangar et al. [34] highlighted that the avocado seed, considered an agro-industrial by-product, contains relevant bioactive compounds such as catechins, epicatechins, gallic acid, and procyanidins, with antioxidant activity equal to or greater than commercial extracts. The authors emphasized its potential in food, nutraceutical, and pharmaceutical applications, citing documented hypocholesterolemic, hypoglycemic, and anti-inflammatory effects in preclinical models.

Notably, Quintero-Espinosa et al. [35] demonstrated that phenolic extracts from the peel of the Colinred variety could prevent pesticide (paraquat/maneb)-induced apoptosis in human neuronal cells by blocking LRRK2 kinase phosphorylation, a protein linked to Parkinson’s disease pathogenesis.

Taken together, these data reinforce that avocado and its by-products not only provide essential nutrients but also act as functional sources of biomolecules with broad applicability in health promotion and chronic disease prevention.

### 3.2. Impact on Gut Microbiota and Lipid Metabolism

Several studies have demonstrated that regular avocado consumption modulates gut microbiota composition, with positive downstream effects on cardiovascular health.

The Habitual Diet and Avocado Trial (HAT), a 26-week randomized clinical trial involving adults with abdominal obesity, showed that the daily intake of one whole avocado significantly increased microbial diversity–reflected both α and β indices, and selectively enriched beneficial bacterial genera. Notably, *Faecalibacterium prausnitzii*, a well-known producer of short-chain fatty acids (SCFAs) and modulator of intestinal inflammation, was found in greater abundance following avocado consumption [38].

Similar findings were reported in the *Persea americana for Total Health* (PATH) study, in which adults consumed 140–175 g of avocado daily over a 12-week period. This intervention was associated with a 65% increase in gut microbial diversity, along with significant elevations in short-chain fatty acids (SCFAs); notably, acetate (+18%) and stearate (+70%). Additionally, substantial reductions were observed in primary bile acids, including cholic acid (−91%) and chenodeoxycholic acid (−57%), suggesting meaningful intestinal modulation with systemic metabolic implications [39]. These alterations in gut microbiota have been directly associated with improvements in lipid profiles. A meta-analysis by Peou et al. [40], which included ten randomized clinical trials (n = 229), reported that avocado-enriched diets significantly reduced total cholesterol by approximately 18.8 mg/dL (95% CI −24.6 to −13.1 mg/dL) and LDL cholesterol by about 16.5 mg/dL. Additionally, triglyceride levels decreased by an average of 27.2 mg/dL, reinforcing the hypolipidemic and cardioprotective potential of avocado.

These results were corroborated by Matthan et al. [37], who reported similar reductions in the HAT trial participants, even in the absence of significant weight loss—reinforcing avocado’s functional role independent of body weight changes. A meta-analysis by Peou et al. [40], encompassing ten randomized controlled trials, also confirmed consistent reductions in total cholesterol and LDL levels following avocado-enriched diets, strengthening the evidence for its hypolipidemic effects.

In population-based studies, Pacheco et al. [41] analyzed prospective cohorts totaling over 110,000 participants and found that consuming ≥ ½ avocado per day was associated with a 16% lower risk of cardiovascular disease and a 21% lower incidence of coronary heart disease.

Substituting saturated fat sources with avocado has proven particularly effective in reducing relative cardiovascular risk, as demonstrated by several clinical and epidemiological studies. This dietary modification not only contributes to improved lipid profiles by lowering LDL cholesterol and increasing HDL, but also enhances overall nutrient intake due to avocado’s high content of monounsaturated fats, fiber, phytosterols, and antioxidants. Such findings reinforce the role of avocado as a cardioprotective food within balanced dietary patterns, such as the Mediterranean and DASH diets, and support its inclusion in public health strategies aimed at reducing diet-related chronic diseases [42,43].

Thus, current evidence suggests that avocado acts as a functional agent in modulating the intestinal ecosystem and lipid metabolism through mechanisms involving increased microbial diversity, SCFA production, bile acid regulation, and improved plasma lipid profiles. These effects position avocado as a promising dietary component in cardiometabolic disease prevention strategies [44,45].

Despite these promising findings, significant methodological limitations persist in avocado-related research, which must be considered when interpreting results and planning future clinical applications. Table 2 summarizes the main challenges identified in the literature, including the lack of standardized extraction methods, limited pharmacokinetic data, and the absence of multicenter trials with diverse populations.

## 4. Technological Valorization and Emerging Innovations

### 4.1. Green Extraction of Bioactive Compounds: Technological Advances and Sustainable Valorization of Avocado

The growing demand for sustainable industrial practices has catalyzed the development of green extraction technologies aimed at maximizing the recovery of bioactive compounds from agro-industrial residues, particularly avocado (*Persea americana*) peel and seed. Emerging techniques such as UAE, MAE, and NADES have demonstrated superior performance over conventional methods, offering improved extraction efficiency, selectivity, and environmental sustainability (Table 3). Recent advances highlight the synergistic potential of UAE combined with NADES in enhancing phenolic compound recovery while preserving antioxidant activity under mild conditions [25]. Likewise, MAE has proven to be a rapid and energy-efficient strategy for isolating thermolabile compounds from avocado seed matrices [26,27].

Recent studies have directly compared emerging extraction methods in avocado by-products. Miramontes-Corona et al. (2024) evaluated the phenolic yield and bioactivities of avocado seed extracts obtained via UAE, MAE, and conventional maceration. Their findings revealed that UAE provided the highest extraction efficiency for total phenolic compounds and superior antioxidant activity (DPPH and ABTS), while MAE extracts exhibited notable antimicrobial effects, particularly against *Staphylococcus aureus*. These results underscore the complementary strengths of each technique, highlighting UAE’s suitability for antioxidant-rich formulations and MAE’s potential in antimicrobial applications [26]. Such comparisons reinforce the relevance of green technologies tailored to specific functional targets.

#### 4.1.1. Natural Deep Eutectic Solvents (NADES) and Ultrasound-Assisted Extraction (UAE)

Among the most promising strategies for the green extraction of bioactive compounds from avocado residues, the application of NADES has emerged as particularly advantageous. These innovative solvents are composed of natural constituents such as choline chloride, organic acids, and sugars. They exhibit low toxicity, high biodegradability, and strong solvating capacity for polar and semi-polar metabolites, making them highly effective for extraction of phenolic compounds and other natural antioxidants [48,49].

The efficiency of NADES-based extraction can be further enhanced by combining it with UAE. The acoustic cavitation generated by ultrasound induces controlled disruption of plant cell walls, facilitating solute release and enhancing mass transfer. This synergy allows for faster, more selective extraction with reduced solvent volumes and energy input, in alignment with the principles of green chemistry [50,51].

This synergy has proven particularly effective in the valorization of avocado by-products. Muhammad et al. [46] applied a combination of choline-acetic acid-based NADES with UAE to Hass avocado peel, achieving yields of up to 8.29 mg GAE/g and strong antioxidant activity (FRAP and DPPH), evidencing the functional potential of the resulting extract.

Similarly, Della Posta et al. [52] developed a green extraction system using choline–lactic acid NADES combined with UAE, achieving high efficiency in phenolic release from avocado peel. González-García et al. [25] reported that replacing 70% ethanol with choline–acetic acid-based NADES led to a >35% increase in total phenolic recovery, reaching values of 92.03 mg GAE/g. In addition to quantitative efficiency, improvements in extract stability and greater selectivity for functional compounds were highlighted, reinforcing their nutraceutical applicability [53,54,55].

Therefore, the NADES + UAE combination represents a viable and environmentally sound technological solution for the sustainable use of avocado residues, supporting the development of bioactive ingredients for functional foods, supplements, and natural cosmetics [56,57].

#### 4.1.2. Microwave-Assisted Extraction (MAE)

MAE uses electromagnetic radiation to promote rapid and selective heating of plant matrices, increasing cell wall rupture efficiency and facilitating the release of bioactive compounds. Using polar solvents such as ethanol–water mixtures, MAE provides high yields while preserving thermolabile molecules, making it a sustainable alternative for recovering avocado residues [58,59].

Weremfo et al. [60] applied MAE to *Persea americana* seeds and optimized critical parameters using response surface methodology (RSM). Optimal conditions (58.3% ethanol, 400 W, 4.8 min) resulted in high phenolic and flavonoid extraction, with strong antioxidant activity confirmed through DPPH and ABTS assays—demonstrating superiority over conventional extraction methods.

In a robust study, Del Castillo Llamosas et al. [61] applied microwave-assisted autohydrolysis to avocado seeds, under controlled conditions (150–230 °C, 5 min). This approach yielded extracts rich in phenolics (42.15 mg GAE/g) and fermentable sugars (38.82 g/L of glucose), validating MAE as part of a functional biorefinery model for generating valuable bioactive and fermentable substrates.

Complementarily, Skenderidis et al. [62] explored vacuum microwave-assisted aqueous extraction (VMAAE), a method applied to both avocado peel and seed. The process was optimized using statistical modeling that considered variables such as temperature, time, microwave power, and the ratio between water and sample. This technique resulted in a high total phenolic content (TPC), reaching 0.352 milligrams of gallic acid equivalents per gram of peel per minute and 0.124 milligrams per gram of seed per minute. The antioxidant capacity was confirmed by DPPH IC_50_ values close to 100 milligrams per liter, highlighting the potential of this method for industrial applications.

Together, these studies show that microwave-based techniques—especially when vacuum-assisted—can enhance the sustainable reuse of avocado by-products, generating high-value functional ingredients for food, cosmetic, and pharmaceutical industries.

#### 4.1.3. Combined Techniques (UAE + MAE)

The implementation of combined extraction techniques—specifically, UAE followed by MAE—has shown significant potential for enhancing the recovery and preservation of bioactive compounds from avocado residues. Martínez-Zamora et al. [63] employed response surface methodology to optimize both UAE and MAE for extracting phenolic compounds from avocado peel, demonstrating that UAE achieves superior yield while MAE offers rapid recovery of heat-stable compounds under controlled conditions. This non-thermal and scalable approach resulted in a 54% increase in the recovery of flavan-3-ols and an improvement of 62–76% in antioxidant activity, as measured by DPPH, ABTS and FRAP assays, when compared to conventional ultrasonic bath methods. Microwave-assisted autohydrolysis (MAAH) of avocado seeds has demonstrated multifunctionality by enabling the simultaneous extraction of phenolic compounds and fermentable sugars. Del Castillo Llamosas et al. [61] optimized this process at temperatures between 150 and 230 °C for 5 min, achieving yields of 42.15 mg GAE/g of phenolics and 38.82 g/L of glucose. This selective extraction technique holds considerable promise for integration into biorefinery platforms, facilitating the valorization of multiple bioactive fractions from a single feedstock.

From a technological standpoint, the combination of microwave-assisted fractionation with prior ultrasound treatment improves extraction yields while preserving thermosensitive compounds such as proanthocyanidins, flavanols, and reducing sugars. This integrated, scalable, and efficient methodology supports the comprehensive valorization of avocado by-products for functional applications across diverse industries [63,64].

#### 4.1.4. Industrial Potential and Technological Outlook

The synergistic combination of ultrasound and microwave technologies offers a promising approach to enhance extraction efficiency while preserving the structural integrity of bioactive compounds from avocado residues. Razola Díaz et al. [65] optimized UAE using a sonotrode and ethanol–water solvent systems, achieving a 54% increase in flavan-3-ol content and a 62–76% enhancement in antioxidant capacity. These results highlight the rapid, scalable, and non-thermal nature of this integrated extraction process, underscoring its potential for industrial application. In parallel, MAAH applied to avocado seeds by Del Castillo Llamosas et al. [61] yielded high-value multifunctional extracts—42.15 mg GAE/g of phenolics and 38.82 g/L of fermentable glucose—highlighting its suitability for sustainable biorefinery applications.

Additionally, Skenderidis et al. [62] confirmed the potential of vacuum microwave-assisted aqueous extraction (VMAAE) for industrial-scale extraction of total phenolic compounds (TPCs) from peel and seed, using optimized operational parameters and achieving significant antioxidant activities (IC_50_~100 mg/L). These technological approaches not only promote concentrated antioxidant and sugar-rich extracts but also offer clear advantages: process times under 15 min, reduced energy demand, and improved thermal stability of bioactives. Compared to conventional extraction techniques, VMAAE provides shorter extraction times, greater energy efficiency, and enhanced preservation of thermolabile bioactives, making it a promising approach for large-scale applications.

In summary, the integration of green strategies—including MAE, UAE, and microwave-assisted autohydrolysis (MAAH)—offers a robust and sustainable platform for biorefinery development. This combined approach supports circular economy principles by valorizing avocado peel and seed, which are traditionally considered waste, into high-value functional ingredients for food, pharmaceutical, and cosmetic sectors.

**Figure 2:**
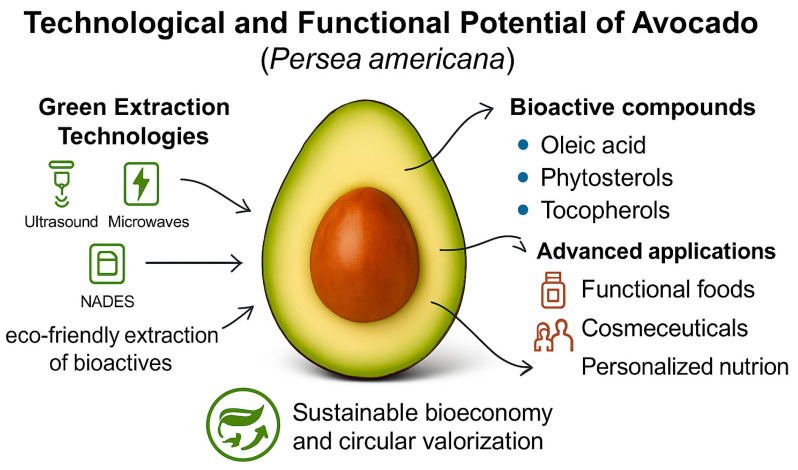
Technological and functional potential of *Persea americana*. Green extraction methods (UAE, MAE, NADES) enable sustainable recovery of bioactives for applications in food, cosmetic, and personalized nutrition sectors.

A visual summary of the green extraction strategies, principal bioactive compounds, and advanced applications in avocado is provided in Figure 2.

## 5. Industrial Applications and Product Development

The increasing demand for functional, safe, and sustainable food products has intensified interest in agro-industrial by-products as a source of novel ingredients. In the case of avocado (*Persea americana*), various technological routes have been explored to transform residues, such as peel, seed, and surplus pulp, into high value-added compounds, with direct application in the food, cosmetic, and nutraceutical sectors. This section presents recent advances in the industrial utilization of avocado derivatives, with particular on innovative formulations that capitalize on their bioactive composition, functional attributes, and technological potential.

### 5.1. Food Applications: Fat Substitutes, Sauces, Snacks, and Functional Oils

Avocado oil, primarily obtained from the pulp, is highly valued for its elevated smoke point (>250 °C), favorable lipid profile, and particularly its high oleic acid content (~70–75%), alongside tocopherols and phytosterols.

These characteristics contribute to both thermal stability and cardioprotective potential. In a study by Unlu et al. [30], the incorporation of avocado oil into salads significantly enhanced the absorption of liposoluble carotenoids—such as lutein, β-carotene, and zeaxanthin—by 7- to 15-fold. This biofunctionality reinforces the role of avocado oil as an effective vehicle for lipophilic bioactives in culinary preparations and functional food formulations.

The use of avocado purée as a partial fat substitute in baked goods has emerged as a nutritionally and technologically advantageous strategy. Othman et al. [4] investigated the replacement of up to 50% of butter with avocado purée in reduced-fat muffins. The reformulated products exhibited a significant decrease in total fat (from 9.7 g to 7.9 g per serving) and saturated fat (from 5.5 g to 4.2 g; *p* < 0.05), without negatively affecting sensory attributes such as taste, texture, or overall acceptability. Moreover, an approximate 16% increase in monounsaturated fatty acids was observed, contributing to a more favorable lipid profile.

In agreement with these results, Othman et al. [4] reported that replacing up to 50% of butter with avocado purée in muffins maintained product weight, height, and sensory acceptance comparable to control formulations. This reinforces the technological feasibility of avocado purée as a partial fat substitute in baked goods, offering a nutritionally advantageous reformulation strategy without compromising consumer acceptance.

Supporting these findings, Othman et al. [4] demonstrated that replacing up to 50% of butter with avocado purée in low-fat muffins resulted in significant reductions in total and saturated fat contents, with no adverse effects on sensory attributes. Similarly, Das and Das [66] conducted a comprehensive review on saturated fat replacement strategies in baked goods and highlighted avocado purée as an effective substitute for butter, contributing to improved lipid profiles while preserving structural integrity and consumer acceptance.

The application of avocado by-products in the food industry has enabled the development of functional ingredients with enhanced nutritional and technological characteristics. Notable innovations include enriched sauces and emulsions, seed-based functional snacks, and nutraceutical-grade oils. For instance, Razola-Díaz et al. [65] optimized flavan-3-ol extraction from avocado by-products using sonotrode ultrasound-assisted extraction, enhancing antioxidant potential. Moreover, Unlu et al. [67] reported that the addition of avocado or avocado oil to meals significantly improved the bioavailability of liposoluble carotenoids, reinforcing its functional role in culinary formulations.

In the emulsions segment, Guzmán-Gerónimo et al. [6] developed a functional mayonnaise using avocado pulp and oil processed by ultrasound. The formulation exhibited significantly reduced microbial load without thermal pasteurization, stable coloration, and high sensory acceptance in preference tests. Sonication proved to be an effective non-destructive technology for ensuring physicochemical stability and microbiological safety in food emulsions. Additionally, Surin et al. [68] demonstrated the potential of avocado seed oil as a sustainable alternative in mayonnaise prototypes, highlighting its antioxidant properties and suitability for clean-label applications. Regarding seed valorization, Permal et al. [7] applied friction-cooking extrusion to produce functional snacks enriched with avocado seed flour. The resulting extrudates exhibited significantly higher total phenolic content and antioxidant capacity compared to commercial brown rice and barley snacks. Additionally, the products contained safe levels of antinutritional compounds such as persin and amygdalin, ensuring industrial feasibility and consumer safety. Moreover, Siol and Sadowska [69] reported that avocado seed powder exhibits high phenolic content, dietary fiber, and physicochemical stability, highlighting its potential as a sustainable, clean-label ingredient for functional food formulations. These strategies reinforce the technological and functional potential of avocado by-products as ingredients for advanced food applications, focusing on health promotion, innovation, and sustainability.

These strategies reinforce the technological and functional potential of avocado by-products as ingredients for advanced food applications, focusing on health promotion, innovation, and sustainability. Recent studies have further expanded this scope. Garofalo et al. [70] demonstrated that avocado pulp can successfully replace pork fat in spontaneously fermented salami, resulting in improved microbial dynamics and oxidative stability. Viola et al. [71] reported the use of Hass avocado seed flour in sourdough semolina bread, enhancing fiber content and antioxidant activity without compromising sensory quality. Nyakang’i et al. [72] explored the incorporation of avocado peel and seed extracts into various food systems, such as baked goods and beverages, contributing to functional enrichment. Additionally, Sarinho et al. [73] developed stable oil-in-water emulsions based on avocado oil using xanthan–guar gum blends, confirming its potential in clean-label emulsified products through detailed rheological and kinetic assessments.

### 5.2. Cosmetics and Dermocosmetics: Avocado Oil and Topical Antioxidants

Avocado oil (*Persea americana* Mill.) has been extensively studied for its favorable lipid profile and richness in bioactive constituents. It consists predominantly of oleic acid (approximately 60–70%), along with linoleic and palmitic acids, and contains substantial amounts of tocopherols (vitamin E), carotenoids (e.g., lutein and β-carotene), and phytosterols [8]. This unique composition imparts emollient, antioxidant, anti-inflammatory, and skin barrier-repairing properties, supporting its broad use into moisturizing, healing, and anti-aging cosmetic formulations. Supporting these findings, Marra et al. [74] emphasize that both avocado oil and its by-products possess great potential for use in cosmeceuticals and functional foods due to their richness in anti-inflammatory and antioxidant bioactive compounds. The study advocates for the full utilization of the fruit as a sustainable strategy for developing health-promoting products.

In a narrative review, Dewi et al. [75] analyzed studies published between 2018 and 2023 and found that formulations containing 3–20% avocado oil—particularly in emulgel systems—improved skin elasticity, reduced dermal inflammation, and provided photoprotective effects. These outcomes are attributed to the synergy among unsaturated fatty acids, tocopherols, and carotenoids in modulating oxidative and inflammatory responses in the skin.

Although clinical trials evaluating exclusively topical application are limited, daily oral administration of avocado or avocado oil in randomized studies has shown systemic benefits. Unlu et al. [66] and Henning et al. [76] observed significant improvements in skin firmness and elasticity, as measured by cutometry after eight weeks of consumption, suggesting translational potential for topical use.

## 6. Future Trends and Research Opportunities

### 6.1. Bioprospecting of Native Cultivars and Stress-Resistant Genotypes

The bioprospecting of native cultivars and stress-resilient genotypes of avocado (*Persea americana*) represents a promising strategy in agri-food research, with important implications for food security, sustainable production system, and the development of novel functional ingredients. While the Hass cultivar currently dominates international markets, numerous regional and wild varieties remain largely underexplored despite demonstrating distinct nutritional and phytochemical profiles, along with enhanced adaptability to abiotic stressors such as drought, salinity, and acidic soils [47].

Studies conducted across tropical and subtropical regions of Latin America, Africa, and Asia have identified genotypes with high levels of bioactive compounds—including phenolics, carotenoids, and phytosterols—as well as superior agronomic performance in agroecological systems and greater resistance to adverse environmental conditions [26,27,28,29,30,31,32,33]. The broad genetic diversity present in traditional and wild germplasms is a valuable reservoir for developing new cultivars with optimized nutraceutical properties and specific industrial applications.

Beyond morphophysiological variation, the integration of omics tools—such as genomics, transcriptomics, and metabolomics—has enabled deeper molecular characterization of promising germplasms, facilitating the mapping of genes associated with biotic and abiotic stress resistance and the biosynthesis of bioactive compounds. In a comprehensive study of 95 Chinese germplasms, wide variation in lipid composition (2.2% to 16.6%) was observed, with several genotypes rich in beneficial fatty acids, amino acids, and essential minerals, 14 of which were identified as functionally superior to the Hass cultivar [38].

Complementarily, Zheng et al. [77] combined transcriptomic and metabolomic analyses to investigate avocado’s response to heat stress, identifying significant changes in abscisic acid signaling, cell wall biosynthesis, and antioxidant pathways. These integrated approaches not only elucidate the molecular basis of environmental adaptation but also reveal metabolic routes associated with the biosynthesis of phytochemicals with therapeutic and functional potential.

Such findings underscore the relevance of systematic bioprospecting and the application of omics technologies as key tools for sustainable genetic improvement, especially in the context of climate change and the growing demand for natural, high-value functional ingredients.

### 6.2. Avocado as a Source of Bioactive Compounds in Pharmaceutical Formulations

Avocado (*Persea americana*) has emerged as a strategic source of bioactive compounds with pharmacological potential, especially due to its composition rich in unsaturated fatty acids, tocopherols, phytosterols, carotenoids, and phenolic compounds. These constituents exhibit anti-inflammatory, antioxidant, wound-healing, hypolipidemic, and immunomodulatory properties, making them attractive for topical, oral, and cosmeceutical formulations with therapeutic purposes [13,23].

From a pharmaceutical technology standpoint, avocado oil has been extensively studied as both a functional excipient and active compound in topical formulations, including healing gels, anti-inflammatory creams, and controlled-release systems. In a recent animal model, Paramanandi et al. [39] demonstrated that a cream containing 15% avocado oil accelerated wound healing in mice. Histological analyses using hematoxylin–eosin and Masson’s trichrome staining revealed a significant reduction in inflammatory cell infiltration on days 3 and 6, as well as a statistically significant increase in collagen fiber density by day 6 with 15% oil (*p* < 0.05) and by day 9 with 10% oil (*p* < 0.05), compared to the control group. These findings reinforce the potential of avocado oil as a functional ingredient in regenerative and anti-inflammatory topical systems.

The unsaponifiable fraction of the oil, composed mainly of phytosterols such as β-sitosterol, has been linked to reduced inflammation and modulation of plasma cholesterol and is being explored in nutraceutical formulations targeting dyslipidemia [23]. Additionally, phenolic compounds present in the seed have shown high antioxidant activity and potential as adjuvants in anti-hyperglycemic and hepatoprotective therapies [13].

At the frontier of pharmaceutical innovation, techniques such as nanoemulsions, lipid nanocapsules, and liposomes containing avocado oil are being investigated to enhance the bioavailability, oxidative stability, and cutaneous penetration of bioactives. In a clinical study, Henning et al. [76] reported that daily avocado consumption by adult women over eight weeks led to significant improvements in skin firmness and elasticity, potentially related to the lipophilic antioxidant content (e.g., tocopherols and lutein) in the pulp.

Despite such advances, the literature still lacks standardization of extracts, pharmacokinetic assessments (ADME), and robust clinical trials to validate the therapeutic efficacy of avocado-derived compounds in humans. Studies integrating omics approaches, mass spectrometry, and pharmacokinetic modeling are essential to enable the regulated use of avocado-based pharmaceutical inputs.

Therefore, avocado stands out as a strategic raw material for the development of next-generation phytopharmaceuticals and cosmeceuticals capable of meeting the rising demand for natural, safe, and sustainable therapies.

### 6.3. Use in Functional and Personalized Foods (Precision Nutrition)

Avocado (*Persea americana*) has gained prominence as a strategic ingredient in the formulation of functional foods and personalized nutrition strategies, due to its rich and diverse bioactive composition. High in monounsaturated fatty acids (MUFAs), soluble dietary fiber, phytosterols, tocopherols, carotenoids, and phenolic compounds, avocado supports lipid modulation, glycemic control, intestinal homeostasis, and the regulation of inflammatory processes—key aspects in dietary strategies for the prevention and management of noncommunicable chronic diseases [13,23]. In a randomized clinical trial, Henning et al. [37] evaluated 39 healthy women who consumed one avocado per day for eight weeks. The results showed significant improvements in skin elasticity and firmness, along with favorable metabolic changes, suggesting systemic effects mediated by bioactives in the pulp. These interindividual responses were associated with baseline dietary profiles and gut microbiota composition, highlighting avocado’s potential in the context of precision nutrition.

Similarly, the Habitual Diet and Avocado Trial (HAT) conducted by Yang et al. [32] demonstrated that daily avocado consumption for 26 weeks significantly increased gut microbial diversity (α and β indices), with stronger effects in individuals with previously poor-quality diets. Notably, there was an increase in *Faecalibacterium prausnitzii*, a bacterial species associated with short-chain fatty acid (SCFA) production and anti-inflammatory modulation of the intestinal mucosa.

These findings are consistent with previous research on avocado-derived bioactives in other contexts. For instance, Paramanandi et al. [78] demonstrated that topical application of a 15% avocado oil cream significantly enhanced wound healing in mice, reducing inflammatory cell infiltration and increasing collagen density—findings that reinforce the anti-inflammatory and regenerative potential of avocado constituents.

Altogether, these data support avocado’s role as a functional food in personalized nutritional interventions, where genetic, metabolic, behavioral, and microbial factors are considered. Precision nutrition aims to maximize nutrient benefits through individualized dietary recommendations. This model integrates genomics, transcriptomics, metabolomics, and microbiome data to develop more effective prevention and treatment strategies [79].

Moreover, the high content of fermentable fibers and prebiotic compounds in avocado pulp and by-products promotes the growth of beneficial gut microorganisms such as *F. prausnitzii* and *Bacteroides* spp., stimulating the production of SCFAs such as acetate, propionate, and butyrate. These metabolites are directly associated with intestinal barrier integrity and systemic inflammation control. The PATH study conducted by Thompson et al. [39] confirmed that avocado consumption over 12 weeks increased microbial diversity by up to 65%, significantly raised SCFA levels, and reduced inflammatory primary bile acids (e.g., cholic and chenodeoxycholic acids).

In product development, the rising demand for natural and personalized foods has driven the creation of innovative formulations, such as enriched smoothies, encapsulated oils, concentrated powders, and functional bars based on avocado. Encapsulation technologies like nanoemulsions, microencapsulation, and liposomes have been widely used to (i) enhance the bioavailability of lipophilic compounds such as avocado oil, phytosterols, and polyphenols; and (ii) protect sensitive bioactives from oxidation and ensure controlled release for greater functional efficacy [80].

One notable example is the development of nanoemulsions containing avocado oil formulated with soy lecithin and carrageenan, which produced droplets with an average size of ~150 nm, low polydispersity index (PDI < 0.2), and excellent oxidative stability [81].

In microencapsulation, avocado seed extract stored in a cassava starch matrix retained 90% of its phenolic content after 30 days and exhibited controlled digestibility, indicating potential for use in sustained-release functional foods [80].

Beyond improved biopharmaceutical profiles, the use of liposome and microencapsulation technologies in concentrated powders enables the incorporation of bioactives into snacks, functional beverages, and targeted products, e.g., for glycemic control, insulin resistance, or gut health. In this scenario, avocado stands out as a highly versatile functional food ingredient. Its integration into innovative and sustainable technologies places the fruit at the forefront of precision nutrition strategies and functional food development, promoting the intersection of health, sustainability, and technological innovation [79].

## 7. Conclusions

This review consolidates the evidence supporting the multifunctional potential of avocado (*Persea americana*) as a strategic source of bioactive compounds with applications in food, cosmetic, and pharmaceutical sectors. Its diverse phytochemical composition—comprising unsaturated fatty acids, tocopherols, phytosterols, carotenoids, phenolics, and prebiotic fibers—underpins its capacity to modulate metabolic, inflammatory, and cardiovascular pathways, as well as influencing gut microbiota composition.

Technological innovations, including green extraction methods (NADES, UAE, MAE), nanostructured delivery systems, and controlled-release formulations, provide sustainable routes for the valorization of avocado and its by-products. These approaches align with circular economy and green chemistry principles, enabling the design of functional, clean-label products that meet contemporary demands for efficacy and sustainability.

Future research should advance the bioprospecting of native and underutilized cultivars with enhanced resilience and nutritional quality, integrate multi-omics approaches for mechanistic insight, and conduct robust clinical trials to establish safety, bioavailability, and efficacy. Addressing regulatory challenges—particularly the substantiation of health claims—will be essential to promote consumer confidence and facilitate market integration.

From an industrial standpoint, the upcycling of avocado residues (peel, seed, and non-commercial pulp) into standardized functional ingredients offers a tangible opportunity to reduce waste, add value, and expand the bioeconomy. Scaling up green technologies, validating functionality in human studies, and incorporating avocado bioactives into diverse product categories can position *Persea americana* at the nexus of nutrition science, sustainability, and industrial innovation.

## Figures and Tables

**Table 1:** Lipid, phenolic, and mineral composition of different avocado (*Persea americana*) cultivars reported in the scientific literature.

Cultivar/Origin	Pulp Lipids (g/100 g PF)	TPC Peel (mg GAE/g)	TPC Seed (mg GAE/g)	β-Sitosterol (mg/kg PF)	Ca (mg/kg)	Fe (mg/kg)	Source/Reference
Hass (Global reference)	15.0	20–30	30–50	3000	300	10.2	Nasri et al., 2023 [27]
Maluma Hass (Morocco)	30.4	32.5	42.1	4559	532	20.3	Nasri et al., 2023 [27]
Ettinger (Morocco)	29.0	28.4	40.8	4123	517	19.4	Nasri et al., 2023 [27]
Ryan (Venezuela)	18.8	—	—	—	—	—	Gómez-López et al., 2002 [28]
Geada (Brazil)	11.2	23.06	33.03	—	—	—	Oliveira et al., 2022 [29]
Breda (Brazil)	10.4	55.57	83.38	—	—	—	Oliveira et al., 2022 [29]
Chinese germplasms (n = 95)	2.2–16.6	Variable	High variability	Not reported	296–532	8.5–20.3	Liu et al., 2020 [31]

PF: pulp fresh weight; TPC: total phenolic compounds; GAE: gallic acid equivalent.

**Table 2:** Main methodological limitations identified in avocado (*Persea americana*) clinical and experimental research.

Methodological Limitation	Description	Reference
Standardization of extracts and extraction methods	Lack of standardized parameters for extraction (cultivar, ripeness, solvent, temperature). Different methods yield distinct phytochemical profiles.	[46] Muhammad et al., 2024
Poorly investigated bioavailability and pharmacokinetics	Lack of data on absorption, distribution, metabolism, and excretion (ADME). Most studies use non-physiological doses.	[47] Bhuyan et al., 2019
Lack of clinical data on peel and seed	Absence of clinical trials evaluating the efficacy, safety, and bioavailability of bioactive compounds from peel and seed in humans.	[34] Bangar et al., 2022
Simplified methodologies and lack of integrated approaches	Prevalence of conventional antioxidant assays (e.g., DPPH, FRAP); lack of omics-based studies (metabolomics, transcriptomics, microbiota).	[39] Thompson et al., 2021
Limitations in population generalization	Clinical studies involve small, homogeneous groups and short durations. Lack of multicenter trials with robust endpoints and population diversity.	[41] Pacheco et al., 2022

**Table 3:** Comparative analysis of green extraction technologies applied to avocado by-products.

Technology	Advantages	Limitations
UAE	-Enhanced mass transfer and cell disruption-Low temperature and short extraction time-Energy efficient and scalable	-Potential degradation of thermolabile compounds with prolonged exposure-Requires specific optimization for each matrix
MAE	-Rapid heating and high extraction efficiency-Reduced solvent use-Suitable for thermoresistant compounds	-Risk of overheating and degradation of heat-sensitive compounds-Limited penetration depth in large-scale systems
NADES	-Biodegradable and non-toxic solvents-Tailored polarity for specific compounds-Excellent solubilizing properties for phenolics	-High viscosity may reduce mass transfer -Recovery of target compounds may require additional steps

## Data Availability

No new data were created or analyzed in this study.
